# Foreign Body (Solder) and Reaction to the Foreign Body Presenting As a Cutaneous Tender Tumor: Case Report and a New Acronym to Aid in Recalling the Differential Diagnosis of Painful Skin Lesions

**DOI:** 10.7759/cureus.6955

**Published:** 2020-02-11

**Authors:** Philip R Cohen, Hadas Skupsky, Christof Erickson, Antoanella Calame

**Affiliations:** 1 Dermatology, San Diego Family Dermatology, San Diego, USA; 2 Dermatopathology, Compass Dermatopathology, San Diego, USA; 3 Dermatology, Compass Dermatopathology, San Diego, USA; 4 Dermatology/Dermatopathology, Compass Dermatopathology, San Diego, USA

**Keywords:** calm, cutaneous, dermal, hogs, lesion, painful, skin, solder, tender, tumor

## Abstract

Cutaneous tender tumors manifest as painful dermal or subcutaneous masses. Acronyms, a memory aid created from the initial letters of other words, can be used to assist in recalling a list of conditions. We report the case of a man who presented with a painful lesion on his leg; 15 years earlier, hot solder had embedded beneath his skin at that location. The subcutaneous mass was removed, and his symptoms resolved. Microscopic evaluation of the specimen showed a foreign body reaction to metal. Therefore, after correlating his medical history, clinical presentation, and pathology findings, the diagnosis of a foreign body (solder) and a foreign body reaction to solder, as a cause of the patient’s painful skin lesion, was established. Including our patient’s diagnosis for his painful skin lesion, the list of conditions that have been described as presenting as a cutaneous tender tumor include calcinosis cutis, angioendotheliomatosis, leiomyoma, metastases, hidradenoma, osteoma cutis, glomus tumor, scar, fibromyxoma, leiomyosarcoma, eccrine angiomatous hamartoma, Dercum’s disease, piezogenic pedal papule, eccrine spiradenoma, neurilemmoma, something else (such as foreign body (solder) and a reaction to the foreign body), angiolipoma, neuroma, dermatofibroma, granular cell tumor, endometriosis, thrombus, blue rubber bleb nevus, angioma, chondrodermatitis nodularis helicis, and keloid. We introduce a novel acronym for painful lesions of the skin that was inspired by the book Charlotte’s Web in which a barn spider (Charlotte), by weaving praises of a pig (Wilbur) into her web, is responsible for the pig’s life being spared. Wilbur is a calm pig; however, there was an episode when he temporarily fled his pen and was subsequently induced, with a pail of slop, to get back into the pen. The new acronym for cutaneous tender tumors uses the initial letter of each of the 26 currently established painful skin lesions: CALM HOGS FLED PENS AND GET BACK.

## Introduction

Dermal and subcutaneous lesions can present as tender masses. Although the diagnosis may be suspected based on the clinical history or the location or the morphology of the tumor, a biopsy for pathologic evaluation is often required to establish the diagnosis. However, the presence of pain is a unique feature that aids in allowing the clinician to formulate an appropriate differential diagnosis prior to tissue confirmation [1].

Acronyms have been used as memory aids to assist in listing the potential possibilities for a painful skin lesion; specifically, the first letter of each tumor is used to create either a word or a phrase or a sentence. Glomus tumor, leiomyoma, eccrine spiradenoma, neuroma/neurilemmoma, dermatofibroma, and angiolipoma (GLENDA) and eccrine spiradenoma, neuroma, glomus tumor, leiomyoma, angiolipoma, neurilemmoma, and dermatofibroma (ENGLAND) were early acronyms for cutaneous tender tumors [1-3]. In 1993, Naversen et al. introduced a new acronym for the painful tumors of the skin, which included leiomyoma, eccrine spiradenoma, neuroma, dermatofibroma, angiolipoma, neurilemmoma, endometrioma, glomus tumor, and granular cell tumor: LEND AN EGG [2]. Naversen et al.’s acronym remained unchanged until 2018, when Bhat et al. added blue rubber bleb nevus and tufted angioma to the differential diagnosis of cutaneous tender tumors and modified the acronym for the list of painful skin tumors: blue rubber bleb nevus, leiomyoma, eccrine spiradenoma, neuroma, dermatofibroma, tufted angioma, angiolipoma, neurilemmoma, endometrioma, glomus tumor, and granular cell tumor (BLEND TAN EGG) [3].

We recently performed a comprehensive review of the literature and discovered 25 cutaneous tender tumors; in addition to describing the unique painful dermal lesions of two patients caused by osteoma cutis or an organizing thrombus, we created a new acronym for these lesions, which include calcinosis cutis, angioendotheliomatosis, leiomyoma, metastases, hidradenoma, osteoma cutis, glomus tumor, fibromyxoma, leiomyosarcoma, eccrine angiomatous hamartoma, Dercum’s disease, piezogenic pedal papule, eccrine spiradenoma, neurilemmoma, angiolipoma, neuroma, dermatofibroma, granular cell tumor, endometriosis, thrombus, scar, blue rubber bleb nevus, angioma, chondrodermatitis nodularis helicis, and keloid: CALM HOG FLED PEN AND GETS BACK [1]. However, after our paper was published, we encountered a man with a subcutaneous foreign body (solder) and a reaction to the foreign body that presented as a tender skin lesion; since this lesion was not included in our earlier acronym, we respectfully introduce a minor modification of the acronym: an ‘S’ for ‘something else’ that not only includes the diagnosis of this patient’s painful cutaneous lesion (which is a foreign body (solder) and a reaction to the foreign body) but also allows for the incorporation of new tender skin lesions that may be encountered in future patients. The new acronym includes calcinosis cutis, angioendotheliomatosis, leiomyoma, metastases, hidradenoma, osteoma cutis, glomus tumor, scar, fibromyxoma, leiomyosarcoma, eccrine angiomatous hamartoma, Dercum’s disease, piezogenic pedal papule, eccrine spiradenoma, neurilemmoma, something else, angiolipoma, neuroma, dermatofibroma, granular cell tumor, endometriosis, thrombus, blue rubber bleb nevus, angioma, chondrodermatitis nodularis helicis, and keloid: CALM HOGS FLED PENS AND GET BACK.

## Case presentation

A 74-year-old man presented for the evaluation and treatment of a painful lesion on his right leg of several years’ duration. His medical history is significant for prostate cancer (treated with radiotherapy and leuprolide acetate injections every three months) and hand dermatitis (with patch test confirmed allergic contact dermatitis to four allergens: diazolidinyl urea, methylchloroisothiazinoline/methylisothiazinolone, tixocortol-21-pivalate, and wool alcohol). Fifteen years earlier, his occupation involved soldering; he recalls hot solder dripping onto his right leg and embedding beneath the skin of his right thigh at the same location that corresponds to his pain.

**Figure 1:**
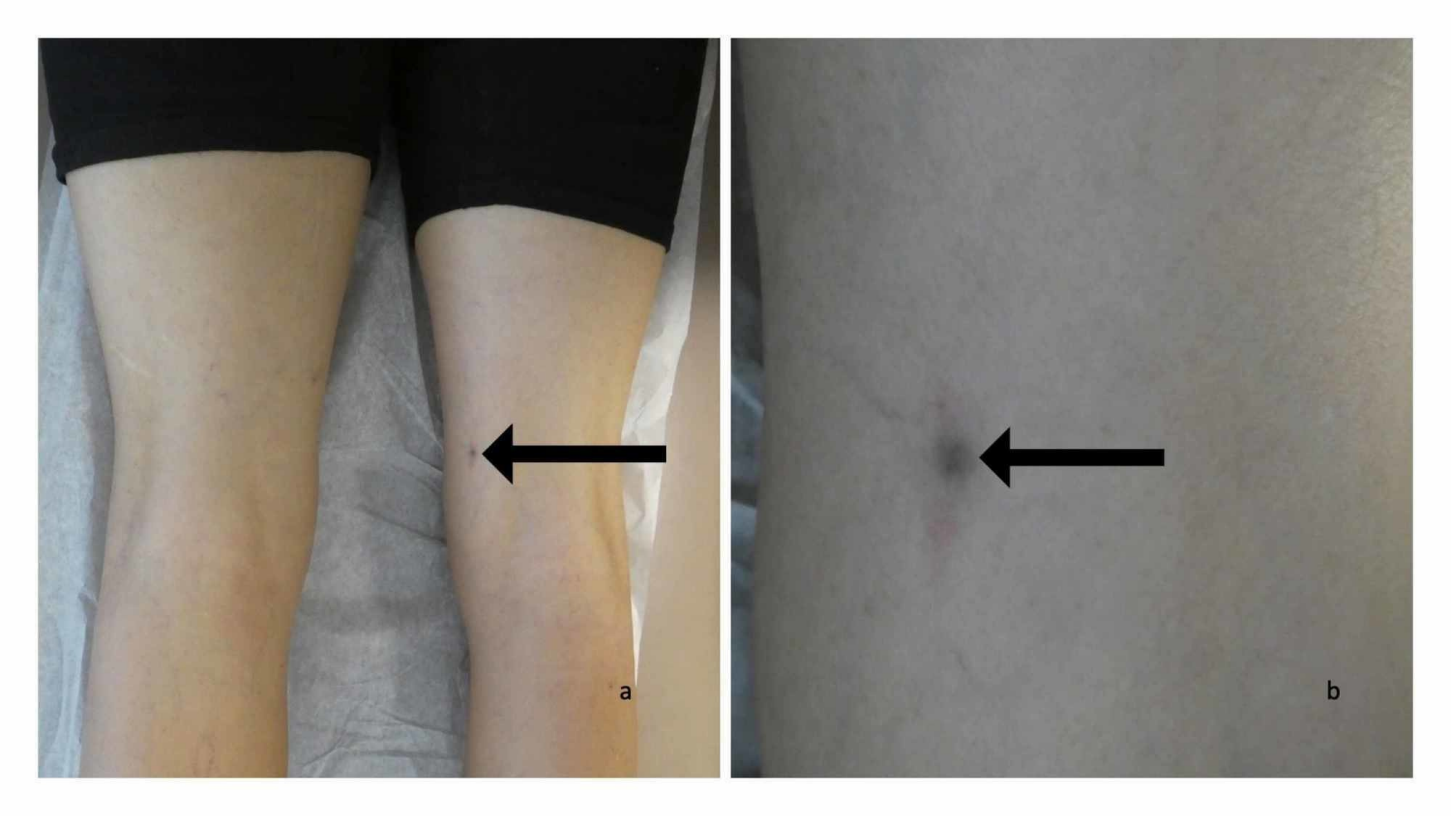
Clinical presentation of foreign body (solder) and foreign body reaction to solder presenting as a cutaneous tender lesion Distant (a) and closer (b) views of the posterior medial right thigh of a 74-year-old man showed a dark brown 5 x 5 millimeter patch surrounded by a lighter tan brown patch (black arrow). It is located at the same site on which hot solder dripped onto his right leg and embedded beneath the skin 15 years earlier. Palpation of the area demonstrates a tender subcutaneous nodule.

Cutaneous examination of his posterior medial right thigh showed a dark brown 5 x 5 millimeter patch. This dark area was surrounded by a lighter tan brown patch. A tender subcutaneous nodule beneath the colored skin was palpable (Figure 1).

**Figure 2:**
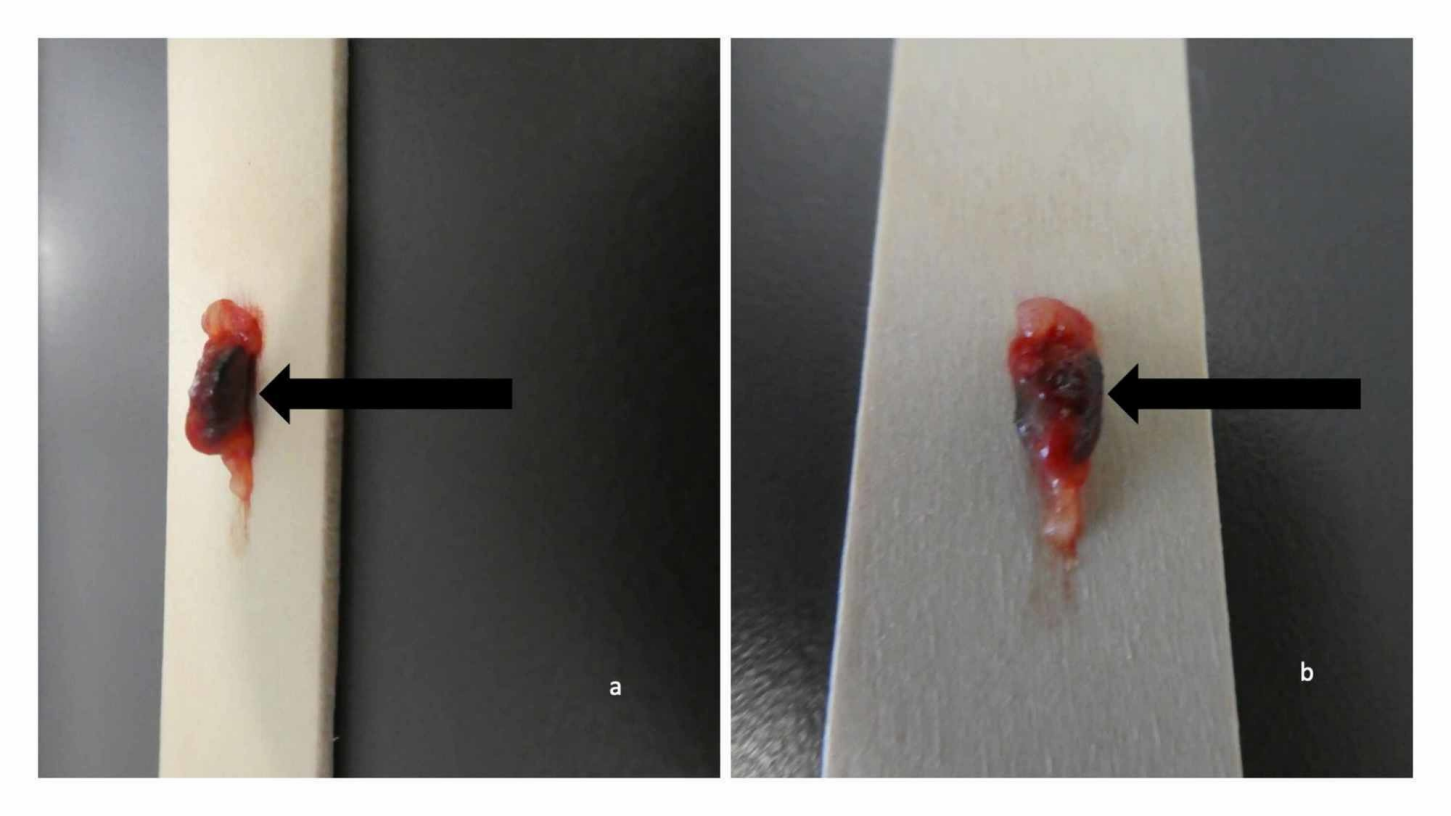
Gross presentation of foreign body (solder) presenting as a painful skin lesion Distant (a) and closer (b) views of a piece of solder (metal alloy) embedded in the subcutaneous fat (black arrow).

A three-millimeter punch biopsy was performed. The 3 x 3 x 5 millimeter cylinder of tissue consisting of epidermis, dermis, and underlying fat was removed. Inspection of the wound demonstrated a solid material in the subcutaneous fat; this was firmly grasped with forceps detached from the adjacent tissue with scissors and removed through the opening created by the biopsy (Figure 2). The biopsy wound was closed using a nylon suture.

**Figure 3:**
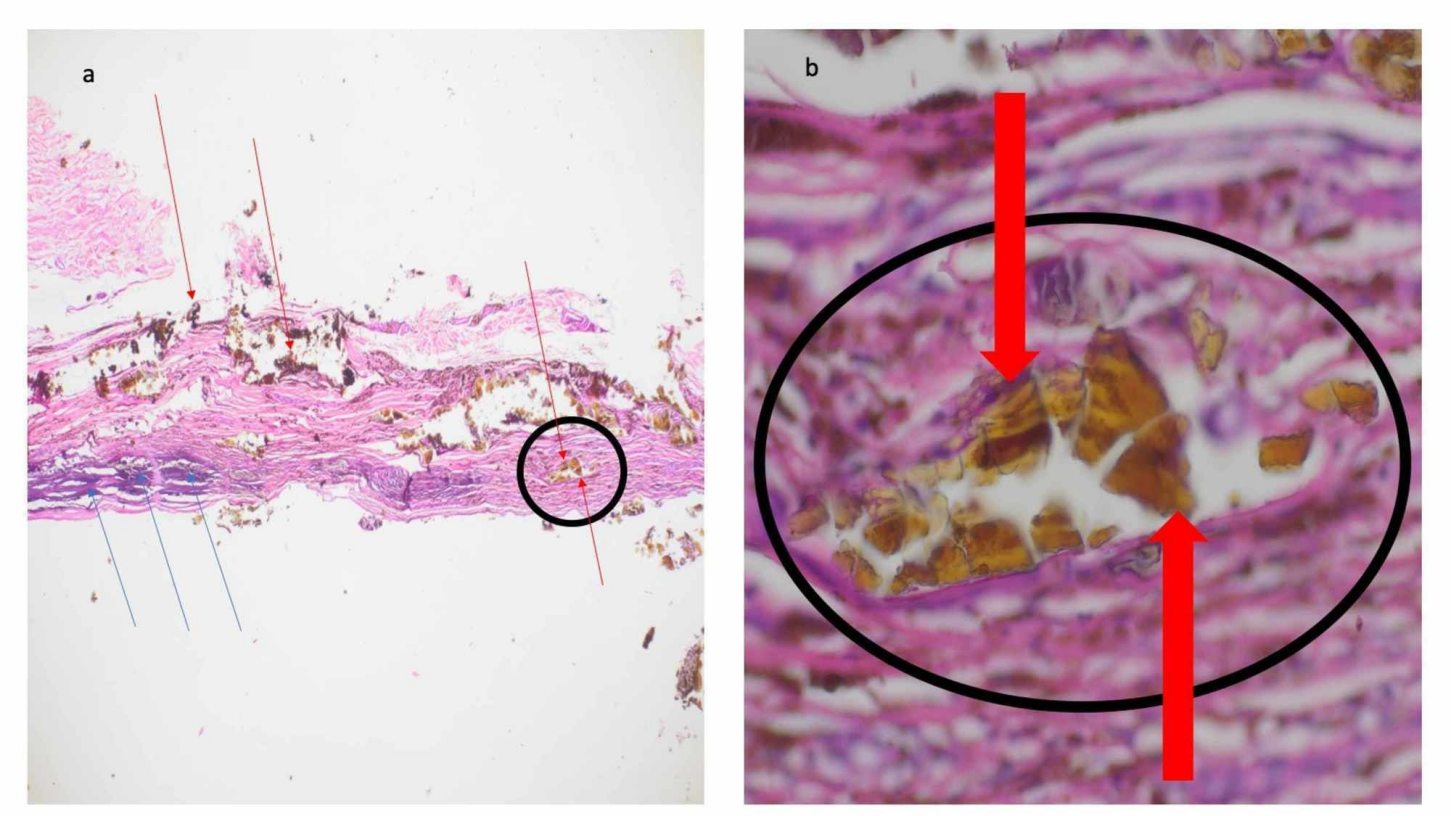
Microscopic presentation of foreign body reaction to solder Distant (a) and closer (b) views of hematoxylin and eosin stained sections of the biopsy specimen from the posterior medial right thigh show an inflammatory infiltrate and foreign body (highlighted within the black circle). There is not only fibroplasia but also an infiltrate of histiocytes and lymphocytes (blue arrows) in the dermis. Foreign material (solder) is present also present in the dermis (red arrows); the metal alloy appeared as brown amorphous masses (hematoxylin and eosin: a, x10; b, x40).

Microscopic examination of the tissue (after removing the solid piece of metal) showed fibroplasia and a mixed inflammatory infiltrate consisting of histiocytes and lymphocytes in the dermis. Foreign material (which polarized), recognizable as metal, was visible within not only the histiocytes but also the dermis. After hematoxylin and eosin staining, the metal appeared as brown amorphous masses (Figure 3).

Correlation of the medical history, the clinical presentation, and the pathology findings established the diagnosis of a foreign body (solder) and a foreign body reaction to solder as the etiology of the patient’s painful skin lesion. The biopsy site healed within two weeks, and the sutures were removed. The patient has had no further episodes of pain at that location.

## Discussion

Painful skin lesions can be categorized by their tissue of origin. They can be adipose (angiolipoma, Dercum’s disease, and piezogenic pedal papule), cartilage (chondrodermatitis nodularis helicis), deposition (calcinosis cutis and osteoma cutis), eccrine (eccrine angiomatous hamartoma, eccrine spiradenoma, and hidradenoma), fibrous (dermatofibroma, digital or superficial acral fibromyxoma, hypertrophic scar, and keloid), infiltration (cutaneous metastases and endometriosis), muscle (glomus tumor, leiomyoma, and leiomyosarcoma), neural (granular cell tumor, neurilemmoma, and neuroma), or vascular (blue rubber bleb nevus, organizing thrombus, reactive angioendotheliomatosis, and tufted angioma) [1]. In addition, similar to the lesion in the reported patient, they can result from an exogenous foreign material that has become implanted beneath the skin.

Solder refers to the low-melting fusible metal alloy used in soldering to create a permanent bond between less fusible metals. Solder usually consists of two or more metals that are combined into an alloy. Lead or cadmium used to be components of solder; more recently, combinations of different metals such as antimony, copper, silver, tin, and zinc are used.

Our earlier acronym was inspired by the book Charlotte’s Web [4]. Charlotte, a barn spider, is responsible for the life of a pig (Wilbur) being spared; she accomplished this feat by weaving praises of him into her web. During the story (chapter 3 titled “Escape”), Wilbur-typically a calm pig-fled his pen; a pail of slop is used to successfully induce Wilbur to get back into his pen [4]. Based on this book, which many children and physicians have read, we created our initial acronym to be used for recalling the differential diagnosis of cutaneous tender tumors: CALM HOG FLED PEN AND GETS BACK [1].

The current acronym (CALM HOGS FLED PENS AND GET BACK) is only a minor modification of our prior acronym (CALM HOG FLED PEN AND GETS BACK) (Table 1) [1]. It utilizes the ‘S’ from ‘GETS’ to create ‘HOGS’ from ‘HOG’ and it adds an additional ‘S’ to ‘PEN’ to make ‘PENS’; indeed, the acronym remains grammatically intact. However, the creation of a new category of lesions - under the heading of ‘something else’ - allows for the inclusion of new diagnoses of painful cutaneous tumors (such as a foreign body (solder) and a reaction to the foreign body) that may be encountered without requiring the creation of another acronym.

**Table 1:** Acronyms for cutaneous painful lesions IL, initial letter ^1^Angioendotheliomatosis refers to reactive angioendotheliomatosis. ^2^Metastases refers to cutaneous metastases; this category also includes B- and T-cell cutaneous lymphoma. ^3^Hidradenoma is also referred to as clear cell acrospiroma, clear cell hidradenoma, eccrine acrospiroma, nodular hidradenoma, and solid-cystic hidradenoma. ^4^Fibromyxoma refers to a digital fibromyxoma, which is known as a superficial acral fibromyxoma. ^5^Leiomyosarcoma refers to cutaneous leiomyosarcoma, a rare malignant smooth muscle tumor that originates either in the dermis or the subcutaneous tissue. ^6^Something else includes a foreign body (such as solder) and a reaction to the foreign body. ^7^Endometriosis refers to cutaneous endometriosis. ^8^Thrombus refers to a cutaneous organizing thrombus, which was originally described as a capillary aneurysm of the skin. ^9^Angioma refers to the tufted angioma variant.

2019 Acronym		2020 Acronym	
IL	Condition	IL	Condition
C	Calcinosis cutis	C	Calcinosis cutis
A	Angioendotheliomatosis^1^	A	Angioendotheliomatosis^1^
L	Leiomyoma	L	Leiomyoma
M	Metastases^2^	M	Metastases^2^
H	Hidradenoma^3^	H	Hidradenoma^3^
O	Osteoma cutis	O	Osteoma cutis
G	Glomus tumor	G	Glomus tumor
		S	Scar
F	Fibromyxoma^4^	F	Fibromyxoma^4^
L	Leiomyosarcoma^5^	L	Leiomyosarcoma^5^
E	Eccrine angiomatous hamartoma	E	Eccrine angiomatous hamartoma
D	Dercum’s disease	D	Dercum’s disease
P	Piezogenic pedal papule	P	Piezogenic pedal papule
E	Eccrine spiradenoma	E	Eccrine spiradenoma
N	Neurilemmoma	N	Neurilemmoma
		S	Something else^6^
A	Angiolipoma	A	Angiolipoma
N	Neuroma	N	Neuroma
D	Dermatofibroma	D	Dermatofibroma
G	Granular cell tumor	G	Granular cell tumor
E	Endometriosis^7^	E	Endometriosis^7^
T	Thrombus^8^	T	Thrombus^8^
S	Scar		
B	Blue rubber bleb nevus	B	Blue rubber bleb nevus
A	Angioma^9^	A	Angioma^9^
C	Chondrodermatitis nodularis helicis	C	Chondrodermatitis nodularis helicis
K	Keloid	K	Keloid

## Conclusions

Cutaneous tender tumors present as painful dermal or subcutaneous masses; a biopsy is necessary to establish the diagnosis. Acronyms are a memory aid formed from the initial letters of other words; they are words, phrases, or sentences that can be used to assist in recalling a list of terms or conditions. Several conditions can be associated with tender cutaneous tumors; we respectfully propose a novel acronym that includes not only the currently established tender cutaneous tumors but also potential future painful skin lesions: CALM HOGS FLED PENS AND GET BACK.
